# Neuronal mitochondrial dysfunction in sporadic amyotrophic lateral sclerosis is developmentally regulated

**DOI:** 10.1038/s41598-021-97928-7

**Published:** 2021-09-23

**Authors:** Tanisha Singh, Yuanyuan Jiao, Lisa M. Ferrando, Svitlana Yablonska, Fang Li, Emily C. Horoszko, David Lacomis, Robert M. Friedlander, Diane L. Carlisle

**Affiliations:** 1 Neuroapoptosis Laboratory, Department of Neurological Surgery, University of Pittsburgh, B400 Presbyterian Hospital, 200 Lothrop Street, Pittsburgh, PA 15213 USA; 2 Departments of Neurology and Pathology, University of Pittsburgh, Pittsburgh, PA 15213 USA

**Keywords:** Amyotrophic lateral sclerosis, Mechanisms of disease, Induced pluripotent stem cells

## Abstract

Amyotrophic lateral sclerosis is an adult-onset neurodegenerative disorder characterized by loss of motor neurons. Mitochondria are essential for neuronal survival but the developmental timing and mechanistic importance of mitochondrial dysfunction in sporadic ALS (sALS) neurons is not fully understood. We used human induced pluripotent stem cells and generated a developmental timeline by differentiating sALS iPSCs to neural progenitors and to motor neurons and comparing mitochondrial parameters with familial ALS (fALS) and control cells at each developmental stage. We report that sALS and fALS motor neurons have elevated reactive oxygen species levels, depolarized mitochondria, impaired oxidative phosphorylation, ATP loss and defective mitochondrial protein import compared with control motor neurons. This phenotype develops with differentiation into motor neurons, the affected cell type in ALS, and does not occur in the parental undifferentiated sALS cells or sALS neural progenitors. Our work demonstrates a developmentally regulated unifying mitochondrial phenotype between patient derived sALS and fALS motor neurons. The occurrence of a unifying mitochondrial phenotype suggests that mitochondrial etiology known to SOD1-fALS may applicable to sALS. Furthermore, our findings suggest that disease-modifying treatments focused on rescue of mitochondrial function may benefit both sALS and fALS patients.

## Introduction

Amyotrophic lateral sclerosis (ALS), characterized by upper motor neuron death in the cerebral cortex and lower motor neurons in the brainstem and spinal cord, results in progressive paralysis, disability, and death^1,2^. In approximately 10% of ALS patients, the disease is inherited, associated with known gene mutations, and classified as familial ALS (fALS)^3^. Ninety percent of cases are sporadic ALS (sALS) with no family history and often unknown etiology^4^. Experimental models recapitulating sALS are limited since most sALS cannot be modeled using genetically manipulated mice. Patient-specific sALS and control human induced pluripotent stem cells (iPSCs) fill this gap. ALS iPSCs derived i-motor neurons exhibit features seen in clinical sALS specimens thus they are useful in vitro sALS models^5,6^

Studies using in vivo and in vitro models and patient tissues report multiple mechanisms contributing to ALS^7^, including dysfunctional mitochondria^8–11^. Motor neurons depend on optimal mitochondrial function to fulfill their energetic requirements. Mitochondrial damage causes insufficient ATP production, with multiple detrimental neuronal consequences^12^ and mediates motor neuron intraneuronal damage and death due to calcium-mediated excitotoxicity, increase in ROS generation, and activates the intrinsic apoptotic pathway^13–16^. SOD1G93A murine studies demonstrated impaired mitochondrial calcium buffering capacity^17^, altering electron transport chain efficiency^18^, diminishing protein import, and reducing complex I activity^19^. Motor neurons generated from other fALS models (TDP43, FUS, C9ORF72, CHCHD10) also had compromised mitochondrial function^11,20–22^. Thus, strong evidence implicates mitochondrial dysfunction in motor neuron degeneration during ALS^23–27^. Notably, these data were primarily generated using fALS models.

There is also evidence of mitochondrial dysfunction in sALS. Assessment of postmortem autopsy tissues demonstrated increased mitochondrial density in sALS spinal cord neurons, perhaps due to smaller neuronal cell bodies^28^. Delic et al. (2018) also suggested impaired mitochondrial function; however extensive analysis was not possible due to limitations of using postmortem tissues. Transcriptome analysis of motor neurons generated from sALS iPSCs suggested mitochondrial dysfunction as a focus^29^. Mitochondrial dysfunction has been investigated in fibroblasts from sALS patients^30,31^, although given the cell-specificity of the disease, the applicability of this data to sALS motor neurons is not clear. Thus, much is still unknown regarding mitochondrial function in sALS motor neurons.

In the context of stem cells, mitochondrial structure and function change with differentiation from pluripotency to mature cell types^32^. IPSCs rely principally on glycolysis^33,34^, As cells shift from pluripotency to differentiated, mitochondrial biogenesis and function also change^35–37^. Differentiation induces mitobiogenesis through peroxisome proliferator-activated receptor gamma coactivator 1-α (PGC-1α) upregulation^36^. Moreover, differentiation leads to hexokinase and lactate dehydrogenase loss during the transition from glycolysis to mitochondrial oxidative phosphorylation^37^. Neuronal homeostasis maintenance during these changes is essential, since perturbations in energy metabolism trigger ALS pathogenesis^38^ and inhibition of oxidative phosphorylation complexes is seen in ALS patient tissues as well as in animal models^39–41^. In cellular models, SOD1 mutation results in loss of mitochondrial respiration while increasing glycolytic flux^42,43^. These studies emphasize the importance of understanding the interplay between differentiation state and energy metabolism in the context of ALS, but such studies have not been done.

Consequently, to better understand mitochondrial function in sALS in the context of motor neuron development, we used iPSCs from sALS patients to compare with fALS patients and controls. We differentiated iPSCs from sALS patients, fALS patients and controls to neural progenitor cells (iNPCs), and iPSC-derived motor neurons (i-motor neurons). We investigated mitochondrial parameters by quantifying reactive oxygen species (ROS), mitochondrial membrane potential (MMP), ATP production and mitochondrial protein import machinery as markers of mitochondrial quality and health. Moreover, we assessed each parameter at every developmental stage (pluripotent, iNPC, i-motor neuron) to investigate the timing of mitochondrial changes in the context of differentiation stage.

## Results

### sALS, fALS and control iPSCs were generated and validated using pluripotency markers

Generation of motor neurons from human iPSCs enables analysis of ALS^5,6^. To initiate the project, we generated three sALS lines and one control line in our laboratory to complement lines available from repositories. Skin fibroblast samples from sALS patients and from a non-neurological controls were collected in the UPMC ALS Center, and reprogrammed with lentivirus carrying KLF4, SOX2, OCT4 and cMYC^44^ to generate iPSCs with classic morphological features of a stem cell colony including small tightly packed cells with low cytoplasmic-to-nuclear volume ratio and tight borders (Supplementary Fig. 1a). An additional three fALS (SOD1 mutant), and two non-neurological control iPSC lines were obtained from the NINDS repository and Cedars Sinai. We confirmed the pluripotency of all nine lines through alkaline phosphatase (AP) staining (Supplementary Fig. 1b) and generation of three germ layers in embryoid bodies followed by immunostaining (Supplementary Fig. 1c). Moreover, karyotyping was determined to check the genetic stability for generated lines (SB006, SB002, SB004, SB008) (Supplementary Fig. 1d). Karyotyping for the NINDS lines (GM23338, ND41865*C; ND35661*E, ND35666*C, SOD1I113T) is available with their online certificates of analysis. To further confirm the pluripotency of all lines before starting mitochondrial experiments, expression of pluripotency markers including OCT4, SOX2, and NANOG were assessed by reverse transcriptase (RT) quantitative polymerase chain reaction (qPCR) assay (Supplementary Fig. 1e). Primary fibroblasts from SB008 were used as a negative control and the H1 and H7 human embryonic stem cell^45^ lines were used as positive controls for qPCR. Values are expressed relative to human ESC line H1 gene expression. All lines demonstrated pluripotent gene and protein expression similar to ESCs. Nuclear pluripotent proteins OCT4, SOX2, and NANOG antibodies demonstrated positive nuclear staining as expected for iPSCs. Cell surface pluripotent markers SSEA4, TRA1-60, and TRA1-81 also demonstrated appropriately localized fluorescence by immunocytochemistry (ICC) (Supplementary Fig. 1f). Thus, the newly generated iPSC lines and the iPSC lines obtained from repositories demonstrated pluripotency and there were no significant pluripotency differences among lines regardless of the source of iPSCs.

### IPSC-derived neural progenitor cells (iNPCs) show early neural markers

Because mitochondrial function is known to change during differentiation^32^, we differentiated iPSCs into NPCs (iNPCs) for analysis. The iPSC lines were differentiated into iNPCs using a standard embryoid body protocol. Bright field images demonstrated classic neural progenitor morphology such as intermediate formation of neural rosettes and morphologically recognizable structures that represent the neural tube containing NPCs, which were selected and allowed to propagate and expand (Supplementary Fig. 2a). In addition to morphology, the quality of the iNPCs was assessed using qPCR and immunocytochemistry (ICC) for neural markers. iNPCs demonstrated increased mRNA expression of NESTIN, SOX1, and PAX6 (Supplementary Fig. 2b), and expressed the early neural proteins NESTIN, SOX1 and PAX6 in iNPCs as compared with undifferentiated parental iPSCs (Supplementary Fig. 2c). All iPSC lines, regardless of source, differentiated efficiently into iNPCs.

### IPSCs-derived motor neurons (i-motor neurons) express gene and protein neuronal markers

Loss of i-motor neurons leads to ALS; thus, to better understand mitochondrial function in ALS, We differentiated iPSCs into i-motor neurons using an embryoid body^30^ protocol with a combination of small molecules to regulate various signaling pathways and generate a highly enriched mature i-motor neuron population (Fig. 1a). Bright field images demonstrate neuronal morphology including reduced cell soma area as compared with iNPCs and membrane spreading, along with extended projections connecting adjacent cells (Fig. 1a). Neuronal morphology was quantified by immunostaining using MAP2 and β3-tubulin, along with qPCR using specific primers for HB9, SMI32 and CHAT also confirmed the formation of i-motor neurons (Fig. 1b). The neuronal marker MAP2 is abundant in the soma while β3-tubulin is visible in the processes. In addition to these general neuronal markers, i-motor neurons were further confirmed by immunostaining for specific markers^46^ such as SM132, HB9 and CHAT (Fig. 1c). For each differentiation, we found that our i-motor neuron population was highly enriched, with 75–80% of neurons in each differentiation demonstrating positive staining for CHAT and HB9 (Fig. 1c). Thus, these cultures are all form highly enriched i-motor neuron culture, regardless of source. Furthermore, although two lines had higher expression of neuronal markers than the other lines, there were no changes that were consistent between groups (control, sALS, fALS) that would confound later analysis.

**Figure ﻿1:**
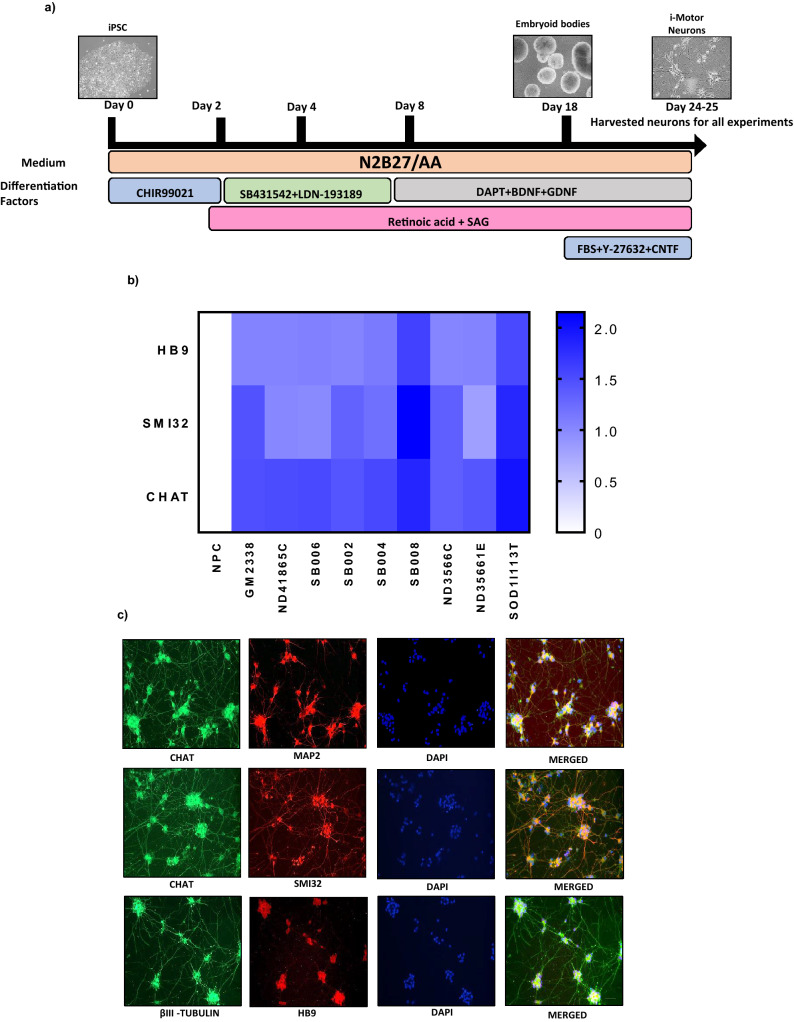
Differentiation of iPSCs to i-motor neurons. (**a**) Representative phase contrast image showing derivation of i-motor neurons from iPSCs to involve the embryoid body stage using small molecules and growth factors. (**b**) qPCR demonstrated expression of motor neuron markers HB9, ChAt and SMI32 in all lines of iPSCs-derived motor neurons. Gene expression was normalized to GAPDH and expressed relative to iNPCs and log10 of fold change values are presented in Heat map generated using Graphpad Prism version 7.03. n = 3 per line, with 3 unique human lines per group., data represent mean of the 3 unique lines +/− SEM. (**c**) Immunofluorescence staining in i-motor neurons shows positive expression for motor neuron markers HB9, βIII-tubulin, ChAT, and SMI32 and were quantified using Image J software.

### sALS- and fALS-derived i-motor neurons have increased reactive oxygen species (ROS) and decreased mitochondrial membrane potential (MMP)

Oxidative stress has been associated with various neurodegenerative diseases including Parkinson’s disease, Alzheimer’s disease, Huntington’s disease and ALS^47^. Oxidative stress and mitochondrial dysfunction are strongly dependent on each other, since most cellular ROS are generated by incomplete reduction of molecular oxygen during oxidative phosphorylation which takes place in mitochondria. To measure the intracellular ROS levels, we used fluorogenic dye 2’, 7’–dichlorofluorescin diacetate (DCFDA) in undifferentiated iPSCs, iNPCs and in i-motor neurons derived from iPSCs. To quantify the change in MMP, a key feature of mitochondrial dysfunction, we measured the MMP using JC1 dye at each stage of differentiation. We found that parental sALS and fALS iPSCs have ROS levels and MMP similar to controls (Fig. 2a(i),b(i)). Similarly, ROS levels were unchanged between groups when they were differentiated into iNPCs (Fig. 2a(ii)). Interestingly, the sALS MMP phenotype begins to emerge in iNPCs, with increased MMP in sALS patients as compared with controls, but not in the fALS iNPCs (Fig. 2b(ii)). An ALS phenotype (both fALS and sALS) is more pronounced after cells are differentiated into i-motor neurons. ROS levels were higher in sALS and fALS i-motor neurons compared with controls as measured by DCFDA (Fig. 2a(iii)). Additionally, MMP, expressed as the ratio of JC1 monomer and aggregates, in sALS and fALS i-motor neurons is significantly decreased in the ALS i-motor neurons compared with control i-motor neurons (Fig. 2b(iii)).

**Figure ﻿2:**
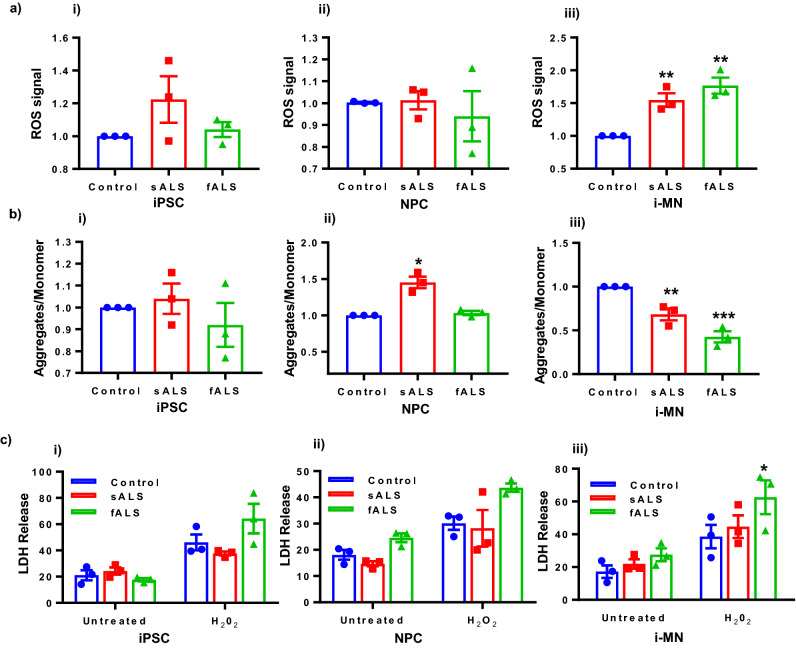
Mitochondrial dysfunction in ALS i-motor neurons. (**a**-i) ROS levels demonstrated that control and ALS iPSCs as well as (**a**-ii) iNPCs have similar level of intracellular ROS, while (**a**-iii) ALS i-motor neurons have increased ROS levels. ROS production was measured using DCFDA dye (excitation = 485 nm; emission = 535 nm) followed by normalization using Hoechst dye for cell number. n = 3 per line, with 3 unique human lines per group. Each data point is the mean value for an individual line relative to a control line, and the bar represents the mean of the three lines +/− SEM, *P < 0.05, **P < 0.01. (**b**-i) MMP quantification shows no difference between control and ALS iPSCs while (**b**-ii) MMP was slightly higher in sALS iNPCs, with no change in fALS iNPCs compared to controls. (**b**-iii) MMP was reduced in ALS i-motor neurons compared with control i-motor neurons. MMP was quantified using JC-1 dye and measured as a ratio of fluorescence of aggregate and monomer forms by plate reader, and expressed as relative fold change compared to controls. n = 3 per line, with 3 unique human lines per group. Each data point is the mean value for an individual line relative to a control line, and the bar represents the mean of the three lines +/− SEM, *P < 0.05, **P < 0.01. (**c**-i) LDH release, an indicator of cell death, demonstrated that control and ALS iPSCs (**c**-ii), iNPCs and (**c**-iii) i-motor neurons have similar cell death rates in response to 24-h incubation of 10 mM H2O2. ALS i-motor neurons have higher cell death rates in response to oxidative stress. n = 3 per line, with 3 unique human lines per group. Each data point is the mean value for an individual line, and the bar represents the mean of the three lines +/− SEM, *P < 0.05. Values were taken using plate reader Synergy H1 from BioTek while statistical analyses were performed with Prism software GraphPad version 7.03.

Unsurprisingly, since each of our lines was generated from a genetically unique patient, we saw variability within our data. Normal technical variability also occurs during directed differentiation of iPSCs into i-motor neuron. To increase the vigor of our data, we differentiated each line three separate times and the data point shown for each line is the mean of these three differentiations. Furthermore, we added complementary assays to quantify ROS and MMP. To measure mitochondrial ROS, we utilized MitoSOX Red reagent, and found elevated mitochondrial ROS in sALS and fALS i-motor neurons (Supplementary Fig. 3a). To complement our MMP JC1 assay, we confirmed the MMP decrease in i-motor neurons using the membrane potential sensitive dye tetramethylrhodamine, methyl ester (TMRM) (Supplementary Fig. 3b).

### Cytotoxicity is elevated in differentiated sALS and fALS i-motor neurons

Mitochondria are key regulators of caspase-mediated cell death^48^ and previous data in a SOD1-mutant mouse model of ALS showed that manipulating this pathway delays motor neuron loss. Some fALS iPSCs are more susceptible to cell death than controls^49^, and SOD1-mutant neurons are highly susceptible to H_2_O_2_-induced cell death due to the reduced ability of SOD to prevent oxidative damage^50^. To quantify cell death, we investigated the susceptibility of sALS undifferentiated iPSCs, iNPCs, and i-motor neurons to H_2_O_2_-induced cell death. We used lactate dehydrogenase (LDH) release as an endpoint for cell death. Toxic stress was induced by exposure of 10 mM H_2_O_2_ for 24 h to each cell type. None of the groups differed in their background (unstressed) cell death rate, and as expected, we found that all groups had increased cell death rates as compared with untreated controls (Fig. 2c(i-iii)). However, LDH release showed that sALS and fALS iPSCs and sALS iNPCs did not differ in vulnerability to H_2_O_2_ as compared with controls. Consistent with previous literature [54], SOD1 mutant fALS i-motor neurons were more susceptible to H_2_O_2_ toxicity than control i-motor neurons. However, this was not true for sALS i-motor neurons (Fig. 2c(iii)). sALS i-motor neurons and control i-motor neurons were equivalent in their susceptibility to death through this mechanism. This suggests although sALS mitochondria have higher levels of intracellular ROS at baseline, their ability to survive when subjected to additional oxidative stress is not compromised.

### Mitochondrial protein import is inhibited in sALS and fALS i-motor neurons

Several studies showed that MMP is essential for the translocation of pre-proteins into the mitochondrial matrix, and MMP reduction may prevent mitochondrial proteins from translocating into matrix^51–53^. To investigate the protein import efficiency in the presence of decreased MMP as demonstrated in Fig. 2, we measured the cleavage of pre-mature (p) ornithine transcarbamylase (OTC) to mature (m) OTC by the mitochondrial processing peptidase upon import into the matrix. This assay quantifies the levels of a classically targeted nuclearly-encoded mitochondrial protein into the matrix over time^54^. Using the pOTC/mOTC mitochondrial protein import assay, we measured protein import into the mitochondria of sALS and fALS iPSCs, iNPCs, and i-motor neurons compared with their respective controls (Fig. 3a,b,c). Mitochondria from sALS and fALS undifferentiated iPSCs had similar mOTC/pOTC ratios at each time point as compared with controls (Fig. 3a). We found a significant difference in the amount of imported protein between mitochondria from fALS and control iNPCs while sALS iNPCs remained unaffected (Fig. 3b). As with MMP and ROS levels, an ALS phenotype fully appears after differentiation into i-motor neurons, and mitochondria from sALS and fALS i-motor neurons had a significantly lower ratios of imported-to-unimported nuclearly encoded protein than mitochondria from control i-motor neurons (Fig. 3c).

**Figure ﻿3:**
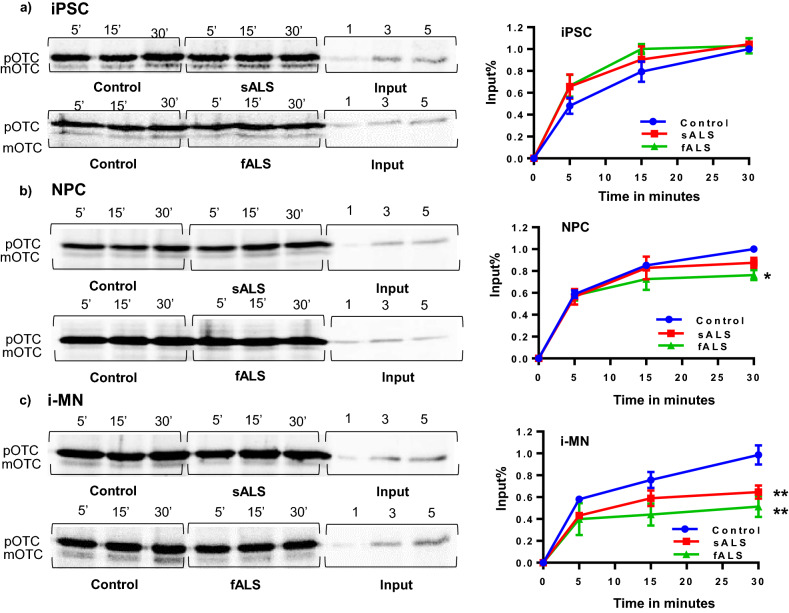
Mitochondrial protein import defect in ALS i-motor neurons. pOTC import assay quantified mitochondrial protein import. (**a**) iPSCs from ALS were unchanged compared to controls. (**b**) iNPCs from fALS have significantly compromised import in comparison to unchanged in control and sALS iNPCs. (**c**) sALS and fALS i-motor neurons decreased pOTC import compared with control i-motor neurons. Mitochondria were isolated using MACS kit, and 25–30 µg of purified mitochondria were used to incubate with [35S]-methionine labeled premature OTC. A representative gel image, cropped to show the bands of interest, along with graph is shown. Protein import is expressed relative to 30 min control for each differentiation stage. n = 3 per line, with 3 unique human lines per group. Each data point is the mean value at that timepoint, relative to import in control at 30 min. +/− SEM, *P < 0.05. Quantified values were taken using Personal Molecular Imger from Biorad while statistical analyses were performed with Prism software GraphPad version 7.03.

### Expression of mitochondrial import machinery proteins decreased in sALS and fALS i-motor neurons

**Figure 4:**
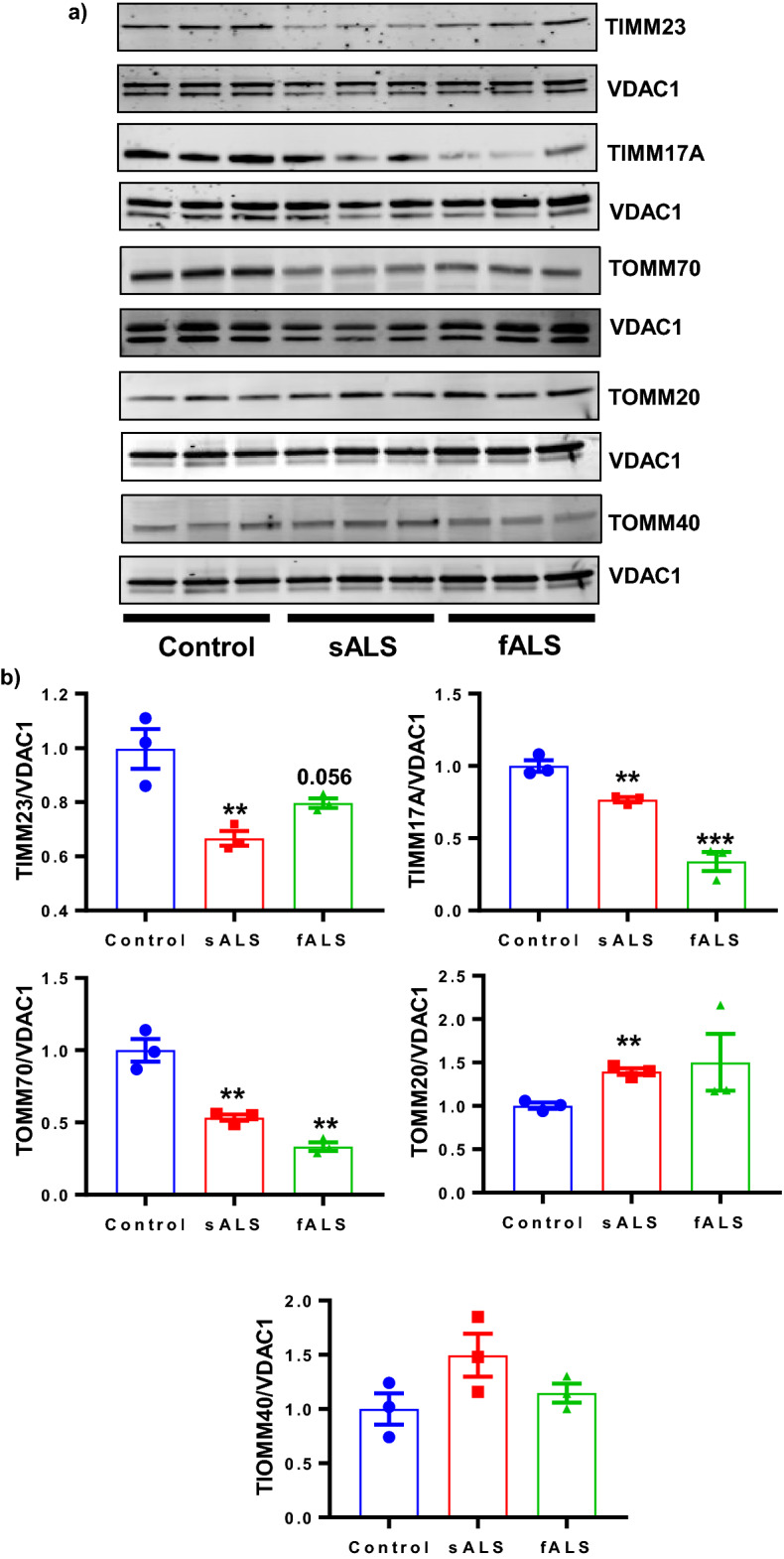
Impaired mitochondrial import complex proteins in ALS i-motor neurons. (**a**) Mitochondrial protein was isolated from i-motor neurons and run on SDS/PAGE followed by immunoblotting to investigate import machinery proteins TOMM70, TOMM40, TOMM20, TIMM23, and TIMM17A. Representative blots for each protein, cropped to show the protein band of interest along with its individual loading control (VDAC1) is shown. (**b**) Densitometry was performed using LiCor Image Studio 2.1 software; n = 3 per line, with 3 unique human lines per group. Each data point is the mean value for an individual line relative to a control line from the same blot, and the bar represents the mean of the three lines +/− SEM, *P < 0.05, **P < 0.01, ***P < 0.001. Statistical analyses were performed with Prism software GraphPad version 7.03.

More than 99% of the mitochondrial proteins are encoded in the nucleus and imported into mitochondria through specified import machineries and these imported proteins are crucial for efficient mitochondrial function^55–57^. Figure 3 demonstrates that mitochondrial protein import is reduced in sALS and fALS i-motor neurons, but the mechanism of this reduction is unknown. One possibility is the previously described decrease in MMP; another is a defect in the import machinery itself. Thus, we investigated the molecular machinery responsible for mitochondrial protein import. Most mitochondrial pre-proteins are imported through the outer mitochondrial membrane translocase (TOMM) and inner mitochondrial membrane translocase (TIMM) protein complexes for proper localization. Since transcriptional defects are well-documented in ALS^58^, we quantified the gene expression of TIMM and TOMM complex members in RNA isolated from i-motor neurons from controls as well as from sALS and fALS patients. There were no consistent changes in mRNA expression for TIMM and TOMM complexes in sALS and fALS lines, with only a decreased in TOMM70 in fALS compare to controls (Supplementary Fig. 4). We also quantified TOMM-TIMM complex protein levels (TOMM70A, TOMM40, TOMM20, TIMM23, and TIMM17A) using immunoblotting of protein extracted from isolated mitochondria obtained from sALS and fALS i-motor neurons as well as controls (Fig. 4a,b). Interestingly, we found that protein levels of TOMM70A, TIMM23, and TIMM17A were decreased in sALS and fALS as compared with control, and there is an increase in TOMM20 in sALS. There were no significant changes in TOMM40 levels among the groups (Fig. 4a,b). Together these data suggest that both the mitochondrial outer membrane protein complex, responsible for import into the intermembrane space, and the inner membrane protein complex, responsible for translocation from the intermembrane space to the matrix, are compromised. Thus, a potential mechanism for decreased mitochondrial protein import is a post-transcriptional defect in translocase protein expression and/or translocation to the mitochondrial membranes.

### Oxidative phosphorylation protein levels decrease in sALS and fALS i-motor neurons

Inefficient mitochondrial protein import can lead to impaired oxidative phosphorylation due to the inability to import to nuclearly encoded proteins that are essential to mitochondrial respiration^59^. Additionally, ROS production was higher in sALS and fALS i-motor neurons than in controls, a common phenotype of impaired oxidative phosphorylation. Impaired oxidative phosphorylation negatively impacts mitochondrial bioenergetics which are required for cell survival. To determine the change in the levels of oxidative phosphorylation proteins, assessment of mitochondrial proteins from sALS and fALS i-motor neurons along with control i-motor neurons were studied using immunoblotting. We find significant changes in proteins representing complex V (ATP5A), IV (MTCO1), III (UOCRC2) and I (NDUFB8) levels in sALS and fALS i-motor neurons compared with control i-motor neurons derived from iPSCs (Fig. 5a,b). Interestingly, protein levels are low for proteins encoded in the nucleus, for which transport is required (ATP5A, UOCRC2, NDUFB8, SDHD), as well as for protein encoded in the mitochondria (MTCO1). This suggests that the oxidative phosphorylation deficiencies are not solely consequences of impaired mitochondrial import.

**Figure ﻿5:**
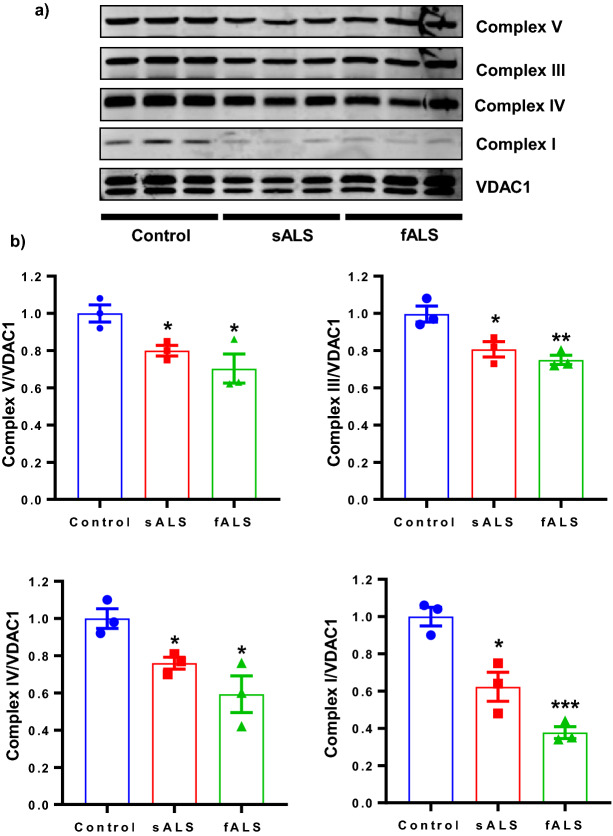
Altered oxidative phosphorylation proteins in ALS i-motor neurons. (**a**) Dysfunction in mitochondrial respiratory chain clearly shown in mitochondrial protein isolated from iPSCs-derived i-motor neurons from ALS lines compared to controls. Representative blots, cropped to show the protein band of interest along with its individual loading control (VDAC1, top band) are shown. (**b**) Densitometry was performed using LiCor Image Studio 2.1 software. n = 3 per line, with 3 unique human lines per group. Each data point is the mean value for an individual line relative to a control line from the same blot, and the bar represents the mean of the three lines +/− SEM, *P < 0.05, **P < 0.01, ***P < 0.001. Statistical analyses were performed with Prism software GraphPad version 7.03.

### Inhibited Mitochondrial Respiration and reduced ATP production in sALS and fALS i-motor neurons

**Figure 6:**
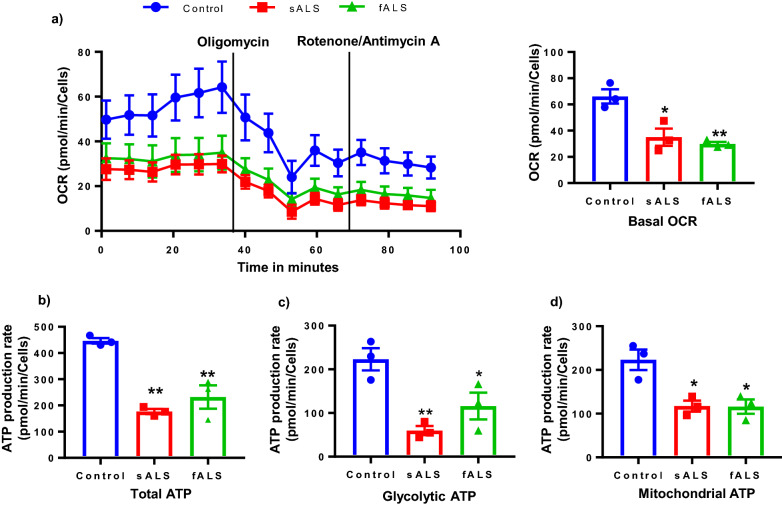
Altered OCR and reduced ATP in ALS i-motor neurons. (**a**) Reduced basal oxygen consumption rate (OCR) in ALS motor neurons is shown. ATP synthase inhibitor oligomycin and mitochondrial complex I inhibitor Rotenone were injected sequentially at the indicated time points into each well after basal rate measurement. Normalization was performed using nuclear stain, n = 3 (**b-d**) ATP production rates quantification by Seahorse XF analyzer in control and ALS motor neurons shows lesser (mitochondrial ATP and glycolytic ATP) in sALS and fALS motor neurons n = 3 per line, with 3 unique human lines per group. Each data point is the mean value for an individual line and the bar represents the mean of the three lines +/− SEM. *P < 0.05, **P < 0.01, ***P < 0.001. Statistical analyses were performed with Prism software GraphPad version 7.03.

Studies report energy metabolism impairment results in ALS progression^60,61^. Relatedly, oxidative phosphorylation is crucial for maintenance of neuronal metabolism and survival. To investigate whether mitochondrial respiration is similarly compromised in both sporadic and familial ALS, we measured ATP production through oxidative phosphorylation and glycolysis using the Agilent SeaHorse Real-Time ATP Rate Assay. Using this assay, we found reduced total ATP production in all fALS and sALS lines compared to control i-motor neurons (Fig. 6a–d). Bioenergetic analysis of mitochondrial respiration demonstrated lower basal oxygen consumption rate (OCR) values in sALS and fALS motor neurons than in control neurons (Fig. 6a, Supplementary Fig. 5), consistent with lower mitochondrial ATP production through oxidative phosphorylation. The assay also quantifies ATP produced through glycolysis. We found that ATP production through glycolysis was also lower in fALS and sALS i-motor neurons than in controls. These data were consistent despite genetic heterogeneity among the sALS and fALS lines used (Supplementary Fig. 5). Therefore, our findings indicated dysfunctional mitochondria accompanied by defective oxidative phosphorylation system that is not compensated through glycolysis.

## Discussion

Mitochondria are a key energy source for eukaryotic cells and neurons are highly dependent upon ATP produced through mitochondrial respiration^62–65^. Disturbances in mitochondrial homeostasis and related mitochondrial activities contribute to pathophysiology of neurodegenerative disease^66^. However, understanding of the mitochondrial defects and their impact on motor neuron survival in sALS, the form of ALS with the highest incidence rate, has lagged due to the inability to genetically model the disease using animals. Newly generated iPSCs from sALS patients have provided opportunities to investigate pathogenic mechanisms in sALS.

In this study, we developed iPSC lines from three sALS patients and one control using reprogramming technology^67^, and obtained an additional five iPSC lines (fALS and controls). Although the lines were made using different strategies from different laboratories, we found consistent results among lines of the same disease status regardless of laboratory of origin. After characterization and confirmation of pluripotency for all iPSC lines, we differentiated them into iNPCs and i-motor neurons using established protocols^68^. A common source of variability in iPSC experiments using unique lines is the expected genetic variability that occurs when using genetically unique patient-derived lines. Additionally, since sALS has, by definition, no known genetic defect, there is no way to generate isogenic (genetically identical except for the mutation of interest) sALS iPSC lines for experimentation. Additionally, technical variability in directed differentiation protocols can add variability to resulting data. In this project, technical variability was controlled for by differentiating each patient-derived line three independent times from pluripotency to the i-motor neuron state for every experiment. The data point representing each line thus is the mean of these three independent differentiations. Most of the remaining variability in the dataset is likely due to the genetic variability that is expected when using nine unique patient lines. While expected, this variability limits the sensitivity of the experiments so that small, but potentially important, changes can not be conclusively identified.

It has long been known that markers of ALS pathology, such as FUS aggregates, are specific to mature motor neurons, but the development of mitochondrial pathology has not been studied. Thus, we included undifferentiated iPSCs, iNPC, and i-motor neurons to investigate mitochondrial health at each developmental stage in the context of sALS and fALS. These three stages model in vitro the in vivo developmental process. To investigate mitochondrial function, we assessed total and mitochondrial ROS, MMP, LDH release during stress, and the mitochondrial protein import rate on all sALS and fALS lines at each stage and compared them with their controls. Among the three developmental stages, only sALS and fALS i-motor neurons were consistently found to be dysfunctional. Undifferentiated iPSCs and iNPCs did not demonstrate consistent defects in these parameters regardless of disease status. These data suggest that the developmental program required to differentiate NPCs into motor neurons may cause qualitative changes in mitochondria that inhibit their normal function. Alternatively, since neurons are much more reliant on oxidative phosphorylation than other cell types, it is possible that low-level inefficiency in mitochondrial function does not cause any overt pathology in undifferentiated ALS iPSCs and NPCs because they rely heavily on glycolysis; however, the high energy demands and reliance on mitochondrial respiration in neurons after differentiation exacerbates the underlying defect leading to measurable pathology in neuronal ALS mitochondria.

Our study also demonstrates the presence of specific mitochondrial defects in sALS i-motor neurons, an understudied area due to the inability to make animal models and also due to the complexity of studying a heterogenous disease. We evaluated mitochondria health using a number of parameters which were previously demonstrated to be impaired in SOD1 fALS i-motor neurons (data that we confirmed using SOD1-mutant patient-specific i-motor neurons as controls in our experiments). Specifically, mitochondrial protein import is impaired in the brains of SOD1-mutant mice^19^. Import of precursor proteins to the mitochondrial inner compartment and MMP are mutually dependent since import requires MMP^69,70^ and maintenance of MMP requires that the mitochondrial function efficiently with a full complement of nuclearly-encoded mitochondrial proteins. Furthermore, MMP deficiency is among the major mechanisms implicated in fALS progression^50,71,72^. In contrast, MMP was increased in sALS fibroblasts^31^. We found that sALS i-motor neuron mitochondria do not efficiently import nuclearly encoded proteins and the mitochondrial membrane is depolarized relative to control i-motor neurons. Thus, sALS MMP defects in motor neurons are not modeled by sALS fibroblasts, which may more closely resemble the sALS NPC phenotype, which also has increased MMP in our study. Instead, the i-motor neuron sALS defect in MMP is similar to that seen in fALS (SOD mutant) motor neurons.

Furthermore, we demonstrate that although sALS, fALS, and control i-motor neurons have equal levels of mRNA transcripts for import proteins, sALS and fALS i-motor neurons mitochondria lack the full complement of proteins responsible for mitochondrial protein import from the cytosol to the intermembrane space and from the intermembrane space to the matrix. This defect may underlie the deficiency in import that we observed in sALS i-motor neurons. The post-transcriptional mechanisms responsible for the decreased mitochondrial protein levels, including possible defects in protein translation, trafficking, and/or insertion into the mitochondrial membranes are unclear and additional studies are needed to understand the contributions of each of these components.

Cellular and animal models having mutated SOD1, CHCH10, or TDP43 genes dysregulate oxidative phosphorylation, which contributes to the ALS phenotype^22,45,50,73^. In sALS motor neurons, we find decreased levels of the major oxidative phosphorylation complexes that were measured (complexes I, III, IV, and V). We find that sALS i-motor neurons, similar to SOD1-mutant i-motor neurons^50^, have increased intracellular ROS levels and are more sensitive to oxidative stress than control i-motor neurons, as demonstrated by increased LDH release after exposure to hydrogen peroxide in culture. Defective mitochondrial respiration and ATP production were observed in various cellular/animal models of SOD1 mutant ALS^40,74–76^. Moreover, markers of cellular respiration were lower in the postmortem spinal cord of sporadic ALS patients^41,77^. The ability to look at human motor neuron energy production using i-motor neurons allows translation of autopsy specimen data to functional assays. However, the conclusions drawn from this data are highly dependent on the assay used. Our data shows that under non-stress conditions, mitochondrial ATP production is decreased in sALS i-motor neurons compared with control. Others demonstrate that respiratory spare capacity is also decreased^61^. Interestingly, our data demonstrate that ATP production through glycolysis is also lower in sALS i-motor neurons than in controls, whereas others show that under respiratory stress when oxidative phosphorylation is artificially shut down, ATP compensation through glycolysis increases^61^. The mechanisms regulating glycolytic ATP production under normal and stress conditions are unclear and warrant further investigation, especially since neuronal function is highly dependent upon maintaining sufficient ATP.

In summary, as a result of these studies, we find a unifying mitochondrial phenotype between sALS and SOD1-fALS i-motor neurons that is specific to mature, terminally differentiated i-motor neurons. Although the initiating etiology is undoubtedly different in sALS i-motor neurons as compared with SOD1-fALS i-motor neurons, the presence of the unifying mitochondrial phenotype suggests that known SOD1-fALS mitochondrial etiology may also apply to sALS i-motor neurons. Furthermore, it also suggests that disease-modifying treatments focused on rescue of mitochondrial function may benefit both sALS and fALS patients. Future studies will determine if the unifying mitochondrial phenotype also applies to fALS initiated by mutations in FUS, VCP, C9orf72, or CHCHD10.

## Materials and methods

### Experimental model and Subject details

#### ALS patients skin biopsies and fibroblast derivation

Human subjects were recruited to this study as approved by the U. Pittsburgh Internal Review Board (IRB protocol 12060073). Skin punch biopsies were taken from non-neurologic control and patient volunteers diagnosed with sporadic ALS (sALS) and kept on ice before culture. Isolated specimens were cut into small pieces and cultured in specific fibroblast media (DMEM supplemented with 10% FBS, 1% penicillin–streptomycin, and 1% L-glutamine). After 1 week, fibroblasts were expanded for cryopreservation and/or reprogramming.

#### Generation of iPSCs

Skin fibroblasts were counted and plated in a dish, and virally transduced with lentivirus carrying KLF4, SOX2, OCT4 and cMYC (purchased from Transgenic and Molecular Core, Magee-Women’s Research Institute) in the presence of 5 µg/ml Polybrene (Sigma, 107,689). The fibroblasts were washed three times with PBS and fed with fresh fibroblast medium after 24 h. 5 days after transduction, fibroblasts were re-plated on a MEFs (irradiated CF-1 mouse embryonic fibroblasts)-coated plate. The next day, fibroblast medium was changed to iPSC medium (KO-DMEM(Invitrogen), supplemented with 20% knockout serum replacement (Invitrogen), 1% L-Glutamine (2 mM), 1% penicillin–streptomycin, 1% MEM Non-essential amino acids, 10 ng/ml of basic fibroblast growth factor (bFGF, R&D). Medium was changed daily for thirty days. IPSC colonies were individually picked and cultured on Matrigel (BD Biosciences) with mTESR1 medium (StemCell Technologies). In total, 4 lines of iPSCs were generated from skin biopsies: 3 lines from patients with sALS (SB002, SB004, and SB008) and 1 line from healthy age-matched subjects (SB006).

#### iPSC Propagation

iPSCs are cultured in StemFlex medium (ThermoFisher) on Matrigel (Millipore). Pluripotent cultures are maintained by manually picking colonies with overt pluripotent morphology (small tightly packed cells, smooth colony borders, low cytoplasmic to nuclear ratio) using a sterile pulled glass pipet under an inverted light microscope into sterile PBS, centrifuged to remove the PBS, and transferred to a new matrigel-coated plate in StemFlex medium. Cultures are expanded before experiments by passaging using ReLeSR (STEMCELL Technologies) as per the manufacturer’s instructions into Matrigel coated plates with StemFlex medium.

#### Karyotyping

Karyotyping was performed at the department of Human Genetics, University of Pittsburgh. We sequenced the sALS lines for C9orf72 gene which is implicated in some cases of sALS, but our lines were found to be negative.

#### Embryoid body formation

Formation of embryoid bodies^30^ was initiated with the dissociation of iPSCs into single cells using Accutase (STEMCELL Technologies). Approximately 1.5 × 10^6^ cells/well were plated in an AggreWell 800™ plate (STEMCELL Technologies), which contained EB formation medium (STEMCELL Technologies) supplemented with 10 μM ROCK inhibitor (Y-27632, Tocris Bioscience) to form uniform embryoid bodies^30^ overnight. The next day, embryoid bodies were transferred to petri dishes and refreshed with embryoid body formation medium every day. After 10 days of incubation, cells were fixed with 4% paraformaldehyde in PBS for immunofluorescence staining.

#### Differentiation of iPSCs into neuronal progenitor cells (iNPCs) and i-motor neurons

iPSCs were differentiated into iNPCs using protocol provided by STEMCELL Technologies. In brief, iPSCs were dissociated using gentle cell dissociation reagent (STEMCELL Technologies) and were grown in STEMdiff neural induction medium (NIM, STEMCELL Technologies) to form neural rosettes. These neural aggregates were later cultured in STEMdiff neural progenitor medium (NPM, STEMCELL Technologies) and passaged in a week to proliferate iNPCs. iPSCs were differentiated to i-motor neurons (also referred to as hiPS-LMN using protocol provided by Bassell laboratory (Holler, 2016), which follows the tenets of neuralization by dual SMAD inhibitors, retinoic acid (RA) induced caudation, ventralization by sonic hedgehog signaling, and maturation using BDNF^46^. Briefly, iPSCs were dissociated using Accutase and grown as EBs cultured in DMEMF12/Neurobasal medium supplemented with N2 and B27 supplements (GIBCO), LDN193189 (200 nM Stemgent), SB431542 (20 µM, STEMCELL Technologies), CHIR99021 (3 µM, STEMCELL Technologies), and ascorbic acid (0.4 µg/mL Sigma) for 2 days. On Day 2 SAG (500 nM, Millipore) and RA (1 µM, Sigma) were added to the medium. On day 4 and 6, CHIR is no longer needed, and the N2B27 DMEM medium for EBs was supplemented with LDN193189 (200 nM), SB431542 (20 µM), ascorbic acid (0.4 µg/mL) SAG (500 nM) and RA(1 µM, Sigma). On day 8, dual SMAD inhibition is no longer required, and neurons were matured by adding neurotrophic factors, brain derived neurotrophic factor (BDNF) (10 ng/mL, Peprotech), glial derived neurotrophic factor (GDNF) (10 ng/mL, Peprotech), and DAPT (STEMCELL Technologies) to the N2B26 medium with SAG (500 nM), RA (1 µM) and ascorbic acid (0.4 µg/mL). This medium was changed every other day until day 18. On day 18, EBs were dissociated into single cells using papain/DNase I (Worthington), filtered through 40-µm strainer and plated on poly-ornithine/laminin coated dishes with day 18 media supplemented with ciliary neurotrophic factor CNTF (10 ng/ml Peprotech). Six days after plating, i-motor neurons were used for experiments. Thus, all i-motor neuron analysis for purity as well as experiments were performed on days 24–28 after the start of differentiation.

#### Immunofluorescence

Cells were fixed in 4% paraformaldehyde in PBS for 15 min, permeabilized with 0.1% Triton X-100 in PBS for 15 min at room temperature and blocked in 20% donkey serum for 45 min. Cells were then washed and incubated in primary antibodies at 4 °C overnight and secondary antibodies at room temperature for 1 h in 0.5% BSA in PBS. Antibodies used were: OCT4, SOX2, NANOG, SSEA-4,TRA-1-60 and TRA-1-81 (Stem Light Pluripotency kit, Cell Signaling, 9656S), SOX1 (R&D, AF3369), Brachyury (Santa Cruz, sc-20109), AFP (Abcam, ab3980), PAX6 (DSHB), Nestin (Millipore, ABD69), HB9 (DSHB,iMNR2), β-III-Tubulin (Cell signaling, D7169), choline acetyltransferase (Abcam, AB143), SMI32 (Covance, SMI-32P), MAP2 (Millipore) and GFAP (Sigma 3893). Secondary antibodies used are Alexa Fluor 488- or 594-conjugated anti-mouse or anti-rabbit IgG (Invitrogen). Antibodies were diluted as per manufacturer’s protocol. Staining for alkaline phosphatase (AP) activity was performed using the alkaline phosphatase live stain kit (Invitrogen, A14353). Full antibody ordering information is listed in Supplementary Table 1. Images were quantified using Image J Software.

#### Analysis of i-motor neuron purity by immunofluorescence

After staining as described above, the total number of β-III-tubulin stained neurons (all neurons) was counted relative to DAPI. The percentage of HB9-stained neurons (specific to i-motor neurons) were also calculated relative to DAPI staining. Assuming 100% of the total neurons (β-III-Tubulin stained neurons), the percentage of i-motor neurons (HB9 stained neurons) was determined. At least 10 separate view fields were imaged per N.

#### Quantitative RT-PCR (RT-qPCR)

RNA was isolated from cultured cells using RNAeasy kit (Qiagen). Reverse transcription PCR (RT-PCR) was done using a high capacity RNA to cDNA kit (Applied Biosystems). Each cDNA product of RT-PCR was amplified and analyzed with Bio-Rad CFX97 Touch and mRNA expression was quantified using the ΔΔCt method with values expressed relative to the mean value of the three unique control lines^78^. The primers used for RT-PCR are listed in supplementary materials. GAPDH was used as an endogenous control to normalize each sample. Each experiment was performed in triplicate (technical repeats) with a biological n = 3 for each cell line.

#### Mitochondria Isolation

Mitochondria were isolated by magnetic sorting using the Mitochondria Isolation kit, human (Miltenyi Biotec) as per manufacturer’s protocol. In brief, 1 × 10^7^ cells were collected and centrifuged at 300 g for 5 min. After PBS washing, the cell pellet was suspended in 1 mL ice-cold lysis buffer and homogenized by passing through a 27 G needle 15–20 times on ice. After homogenization, lysate was incubated with 50 µL of anti-Tom22 MicroBeads in 1 × separation buffer for 1 h at 4 oC. The suspension was then passed through a pre-separation filter (Miltenyi Biotec) on LS column after calibration. After washings with 1 × separation buffer, mitochondria were eluted with IM-2 (Isolation Buffer 2, 225 mM sucrose, 75 mM mannitol, 5 mM HEPES, PH 7.4 at 4 °C) and centrifuged at 13,000 g for 4 min at 4 °C. Isolated mitochondria were kept on ice and used for mitochondrial protein import assay within 2 h after preparation.

#### Immunoblotting

Prior to immunoblotting, mitochondrial protein was quantified using Bradford reagent (Bio-Rad). Mitochondrial protein lysates were prepared with Laemmli buffer along with 10% β-Mercaptoethanol (BioRad). Before running on SDS-PAGE, samples were heated at 55 °C for 10 min. Further, proteins were separated at 160 V in SDS Running buffer (Life Technologies) and transferred to PVDF-FL membrane (EMD Millipore) at 85 V for 85 min. PVDF membranes were then blocked in Blocking buffer (Thermo Scientific) for 45 min at room temperature followed by incubation in desired primary antibodies at 4 °C overnight. Fluorescent IR dye-labeled secondary antibodies (LI-COR Biosciences) were applied for 1 h at room temperature. After incubation, membranes were scanned on Odyssey CLx LI-COR imager to visualize protein bands. Antibodies used: anti-Timm23 (BD), anti-Timm17a (Abcam, ab192246), anti-Tomm40 (Santacruz, SC-365-467), anti-Tomm20 (Abcam, ab56783), anti-Tomm70a (Abcam, ab83841), Total OXPHOS Cocktail mitoprofiler (Abcam, ab110413) and anti-VDAC (Millipore, AB10527, upper band used for quantification) as per manufacturer’s protocol. Secondary antibodies used: donkey anti-goat (LiCor NC9744099), donkey anti-rabbit (Fisher Scientific, 711-055-152), donkey anti-mouse (Fisher Scientific, 715-055-150), donkey anti-goat (715-055-147), goat anti-rabbit (926-32,211, LiCor, 1:20,000); goat anti-mouse (926-68,020, LiCor, 1:20,000). Full antibody ordering information is listed in Supplementary Table 1. Blots were analyzed using upper band of VDAC1 and quantified using LiCor Image Studio 2.1 software.

#### Lactate dehydrogenase (LDH) assay

The extent of cell death was measured in triplicate per n by the LDH assay kit according to the manufacturer’s instruction (Roche Products)^79^. Briefly, supernatant was transferred to a new plate, then the remaining cells were lysed with 1% Triton X-100 for 30 min at 37 °C. Each reaction mixture (100 μl) was added to conditioned media (50 μl) removed from dishes after centrifugation at 2000 × g for 2 min. Absorbance of samples at 490 nm was measured in a plate reader (Synergy H1). The same volume of blank medium was used as the background control. Then, we calculated the slope of LDH reaction of each sample. The cell death rate was calculated as slopesp/(slopesp + slopept), where sp is supernatant and pt is pellet and values were taken using plate reader Synergy H1 from BioTek.

#### Reactive oxygen species (ROS) estimation

Measurement of ROS levels in each cell type was determined using fluorescent dye DCFDA (2′, 7′-dichlorofluorescin diacetate)^80^. In brief, cells were incubated with 20 µM DCFDA for 45 min at 37 °C followed twice by PBS wash. ROS levels were measured after the detection of fluorescent dichlorofluorescein (DCF) at excitation/emission 485 nm/535 nm. For normalization, cells were later incubated with 5ug/ml Hoechst stain for 5 min at 37 °C followed by PBS wash. Cell numbers were measured as per fluorescence signal of Hoechst at excitation/emission 350 nm/461 nm. The relative ROS levels were further calculated as the ratio of DCDFA and Hoechst signals. Mitochondrial ROS level in i-motor neurons was determined by MitoSox dye (Invitrogen) using plate reader. In brief, cells were incubated with 5 µM Mitosox for 15 min at 37˚C followed by PBS wash. After washes, fluorescence was measured using plate reader at excitation/emission 510 nm/595 nm. For normalization, cells were later incubated with 5ug/ml Hoechst stain for 5 min at 37 °C followed by PBS wash. Cell numbers were measured as per fluorescence signal of Hoechst at excitation/emission 350 nm/461 nm. The relative ROS levels were further calculated as the ratio of Mitosox and Hoechst signals were taken using plate reader Synergy H1 from BioTek.

#### Mitochondrial protein import assay

Mitochondria protein import was measured using the human ornithine transcarbamylase (OTC) assay^66^. The OTC precursor cDNA, in pGEM-3Zf(+)-pOTC plasmid, was transcribed and translated in vitro using the TNT-coupled reticulocyte lysate system (Promega) in the presence of L-[35S]methionine (Perkin Elmer). Following translation, [35S] methionine-labeled pOTC was incubated with isolated mitochondria at 30 °C for the indicated times, and mitochondria containing imported OTC were collected by centrifugation (8,000 g for 10 min) and subjected to SDS-PAGE. The radioactive polypeptides on the gel were exposed to a phosphorus screen (GE Healthcare) overnight and were quantified by a personal molecular imager (Bio-Rad PMI system). Data were analyzed as the percentage of mOTC compared to input (total [35S]pOTC amount added to reaction) and by setting the control line at 30 min of import to 1, and expressing all other values as a fraction of the maximum import in the control lines.

#### Mitochondrial membrane potential (MMP) quantification

MMP of cells was measured using membrane permeant fluorescent dye JC-1 (5,5′,6,6′-tetrachloro-1,1′,3,3′-tetraethylbenzimidazolylcarbo cyanine iodide)^80^. Each cell type was incubated with JC-1 for 15 min at 37 °C and washed with PBS followed by estimating the levels of red (excitation 535 nm, emission 590 nm) and green fluorescence (excitation 475 nm, emission 530 nm) using a synergy plate reader. MMP was estimated by red/green fluorescence i.e. aggregates/monomers ratio. To increase the rigor of these conclusions, MMP was also measured using tetramethylrhodamine methyl ester (TMRM)^80^ using plate reader and immunostaining. Cells were incubated with 25 nM TMRM for 30 min at 37 °C and fluorescence were measured using plate reader at excitation/emission 548 nm/573 nm. MMP was also quantified by TMRM normalized with nuclear stain using plate reader Synergy H1 from BioTek.

#### Mitochondrial ATP assay

Total ATP, oxygen consumption rate (OCR) indicating mitochondrial ATP, and glycolytic ATP were analyzed using Seahorse XFe96 Analyzer, Agilent. Seahorse XF Real-Time ATP Rate Assay Kit provided by Agilent was used to detect the ATP production rates from glycolysis and mitochondria simultaneously as per manufacturer’s instructions. Before experiment, the probe plate was hydrated with HPLC grade water in a CO2-free incubator overnight. The XF DMEM assay medium was supplemented with 10 mM glucose, 5 mM pyruvate was kept in a 37 °C CO2-free incubator to maintain the pH value. Then the HPLC grade water in the hydration plate was replaced with calibration solution and kept in a 37 °C CO2-free incubator for an hour. Control, sALS and fALS iPSC-derived motor neurons were seeded into XF96 cell culture microplates (Seahorse Bioscience) at the density of 50,000 cells/well for measurement of OCR and ATP production rates. For the determination of OCR and ATP production rates in sALS, fALS and control i-motor neurons, oligomycin and a mix of rotenone and antimycin A were added according to the manufacturer’s instructions and protocols (Agilent Bioscience).

#### Statistical analyses

Statistical analyses were performed with Prism software GraphPad version 7.03. Data were obtained from at least three independent experiments and expressed as mean ± SEM unless otherwise specified. Specifically, each cell line was differentiated three times to generate three independent biological repeats per line. For group analysis (Control, sALS and fALS), the average value for each line is indicated in each graph. Thus, each independent value in presented graph is the average of at least three data points, and the mean for each group is the mean for the three lines. Each biological repeat has at least three technical repeats. Since all data points are the average value for three independent differentiations, error bars on graphs represent standard error of the mean (SEM). The Student’s t-test for parametric data and paired t-tests were used for experiments with multiple samples from the same source. ANOVA followed by Tukey’s test were used for analysis of more than two groups. *P*-values less than 0.05 were considered statistically significant (indicated in figures as: *P < 0.05; **P < 0.01; ***P < 0.001).

### Ethics declaration

All methods were carried out in accordance with relevant guidelines and regulations.

### Ethics approval and consent to participate

Prior to their participation, all donors of skin biopsies provided their written informed consent and study approval was obtained from University of Pittsburgh Institutional Review Board.

## Supplementary Information

Supplementary Information.
