# Supplementary material for: Neuronal mitochondrial dysfunction in sporadic amyotrophic lateral sclerosis is developmentally regulated
Source: Sci Rep. 2021 Sep 23;11:18916. doi: 10.1038/s41598-021-97928-7 (PMC8460779; doi:10.1038/s41598-021-97928-7)
Supplement: Supplementary file 1 — Supplementary Information. [file 41598_2021_97928_MOESM1_ESM.docx]

**Neuronal mitochondrial dysfunction in sporadic amyotrophic lateral sclerosis is developmentally regulated**

Tanisha Singh^1,3^, Yuanyuan Jiao^1,3,^ Lisa M Ferrando^1^, Svitlana Yablonska^1^, Fang Li^1^, Emily C Horoszko^1^, David Lacomis^2^, Robert M Friedlander^1^, Diane L Carlisle^1#^

**Author Affiliations:**

^1^ Neuroapoptosis Laboratory, Department of Neurological Surgery, University of Pittsburgh, Pittsburgh, PA 15213

^2^ Departments of Neurology and Pathology, University of Pittsburgh, Pittsburgh, PA 15213

^3^Co-first authors Tanisha Singh and Yuanyuan Jiao contributed equally to this manuscript.

^#^Corresponding Author:

Diane L Carlisle PhD

200 Lothrop Street

B400 Presbyterian Hospital

Pittsburgh, PA 15213

412-383-5828

DLC4@pitt.edu

**Keywords:** amyotrophic lateral sclerosis (ALS), mitochondrial dysfunction, mitochondrial protein import, Oxidative phosphorylation, ATP, iPSC, neuronal differentiation, neural progenitors, motor neurons.

**
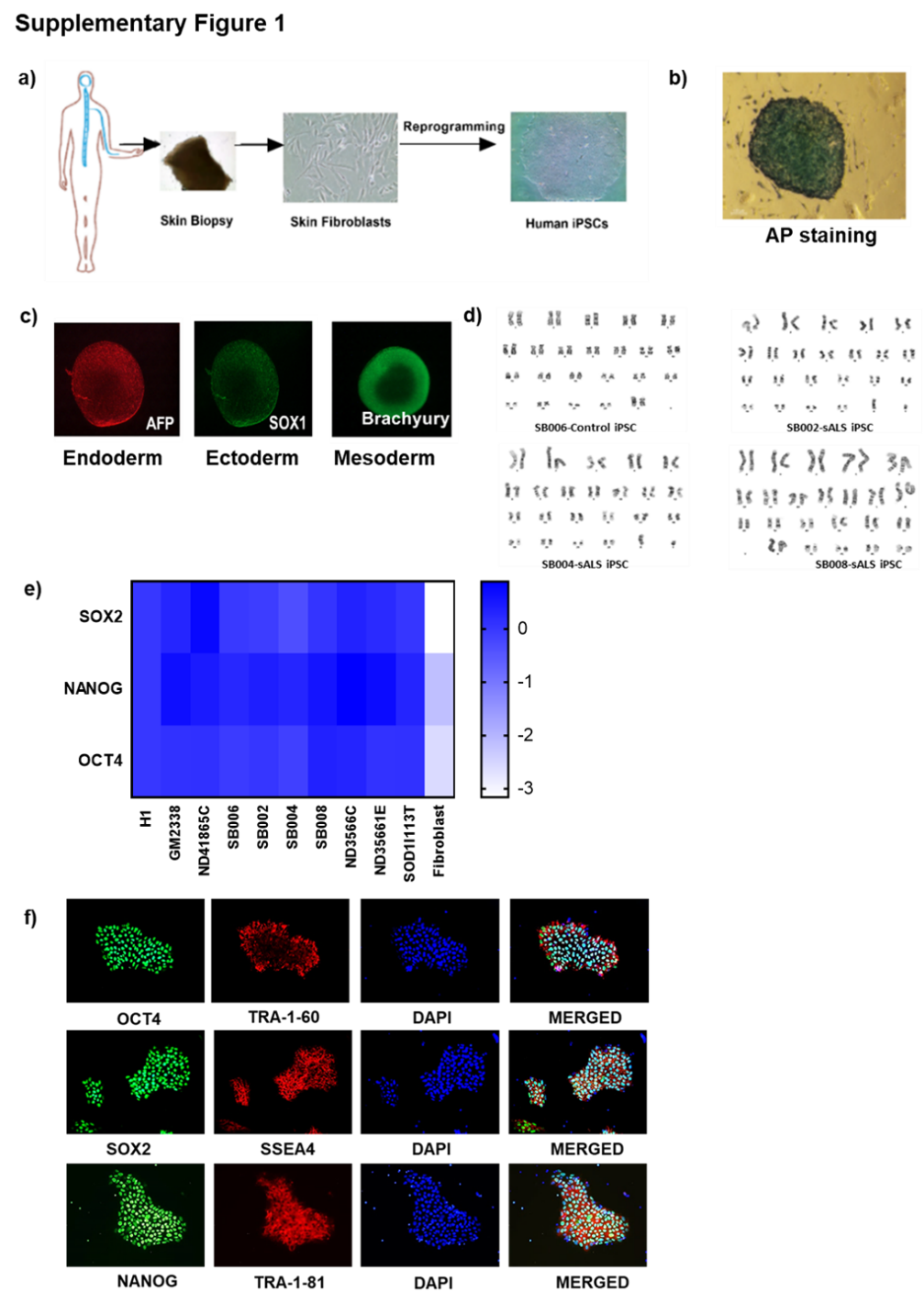
Supplementary Figures:**

**Supplementary Figure 1:** Characterization and confirmation of iPSC lines. (a) Schematic representation of skin fibroblasts that were obtained, grown in culture, and reprogrammed into iPSCs, created using Microsoft Office 365 and original images. (b) IPSCs showed expression of human ESC-specific marker alkaline phosphatase (AP). (c) Immunofluorescence shows expression of molecular markers for the three developmental germ layers by the iPSC EBs: alpha feta protein (AFP) (endoderm), SOX1 (ectoderm), and BRACHYURY (mesoderm). (d) Karyotypes of derived iPSCs were found to be normal. (e) qPCR quantification for pluripotency genes (OCT4, NANOG, SOX2), Gene expression was normalized for loading to GAPDH, and is expressed relative to the H1 ESCs. Log10 of fold change values are presented in the heat map. n = 3 generated using Graphpad Prism version 7.03 and (f) Immunofluorescence for pluripotent markers Oct4, Nanog, Sox2, Ssea3/4, Tra-1-60, and Tra-1-81 and quantified using Image J software.


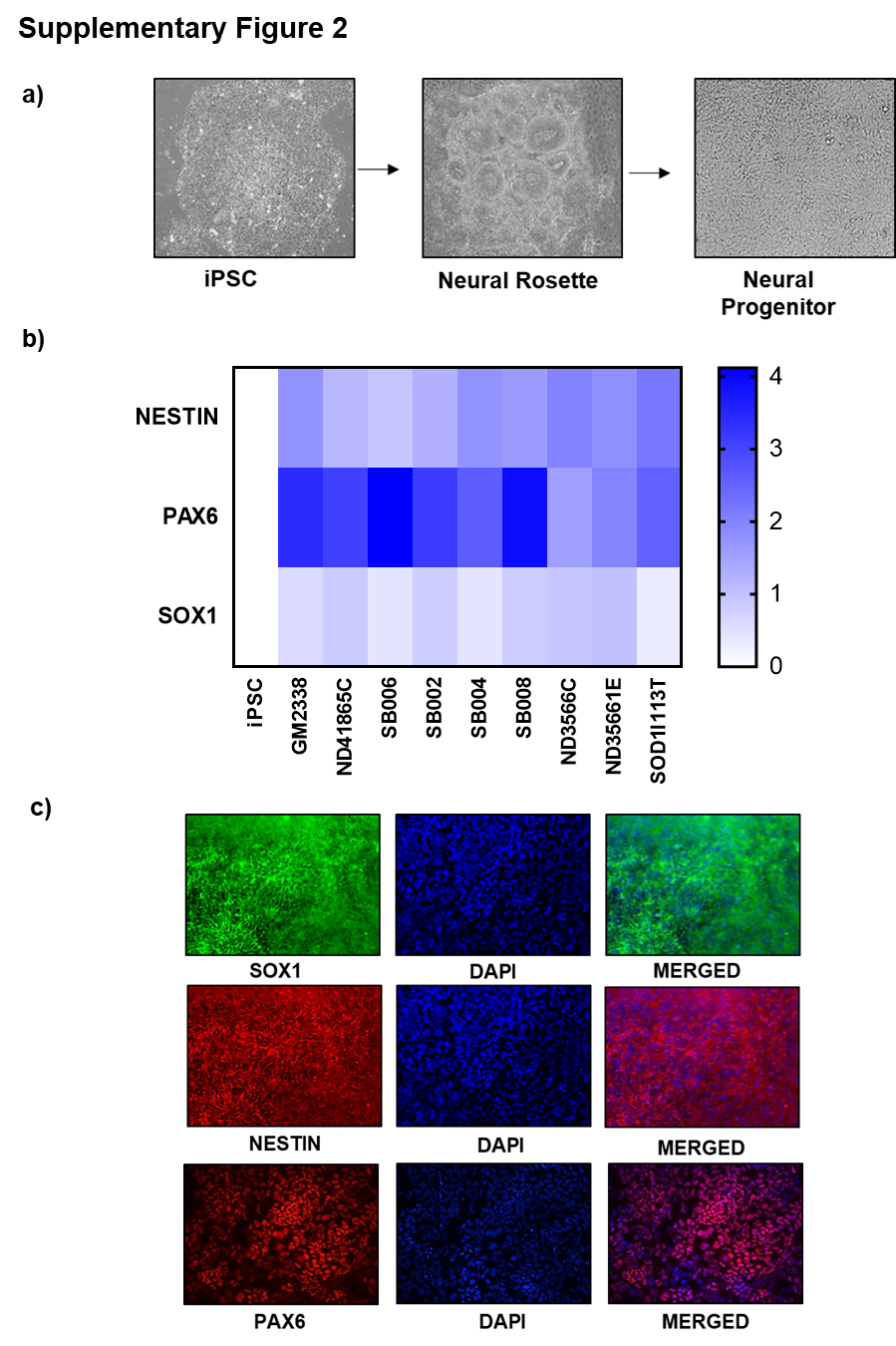


**Supplementary Figure 2:** Differentiation of iPSCs to neural progenitor cells (iNPCs). (a) Representative phase-contrast image showing iPSCs differentiated to neural epithelial cells forming neural tube-like rosettes followed by early neural progenitors. (b) qPCR quantification for the expression of NESTIN, SOX1 and PAX6 in NPCs. Gene expression was normalized to GAPDH and expressed relative to the iPSCs. Log10 of fold change values are presented in the heat map generated using Graphpad Prism version 7.03. n = 3. (c) Immunofluorescence images of iNPCs representing expression of neural markers Sox1, Pax6, and Nestin and quantified using Image J software.

**
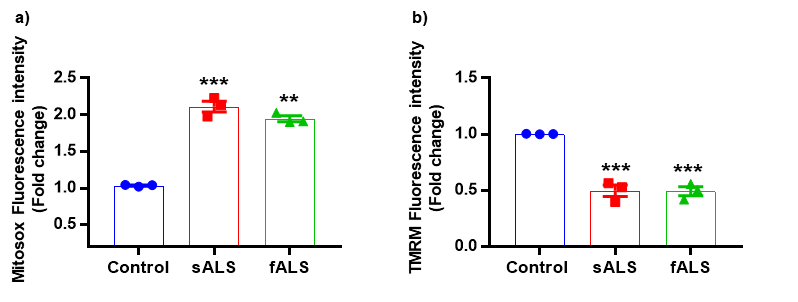
**

**Supplementary Figure 3:** Complementary confirmation of increased ROS and decreased MMP in sALS motor neurons. (a) Increased mitochondrial ROS in ALS i-motor neurons. Quantification of MitoSOX fluorescence indicated ROS that is significantly increased in the ALS i-motor neurons compared with control i-motor neurons. Each data point is the mean value for an individual line and the bar represents the mean of the three lines +/- SEM, *p < 0.05, **p<0.01, ***p<0.001. (b) Decreased mitochondrial membrane potential in ALS i-motor neurons. Quantification of TMRM fluorescence using plate reader indicated MMP that is significantly decreased in the ALS i-motor neurons compared with control i-motor neurons. Each data point is the mean value for an individual line and the bar represents the mean of the three lines +/- SEM, *p < 0.05,**p<0.01, ***p<0.001. Values were taken using plate reader Synergy H1 from BioTek while Statistical analyses were performed with Prism software GraphPad version 7.03


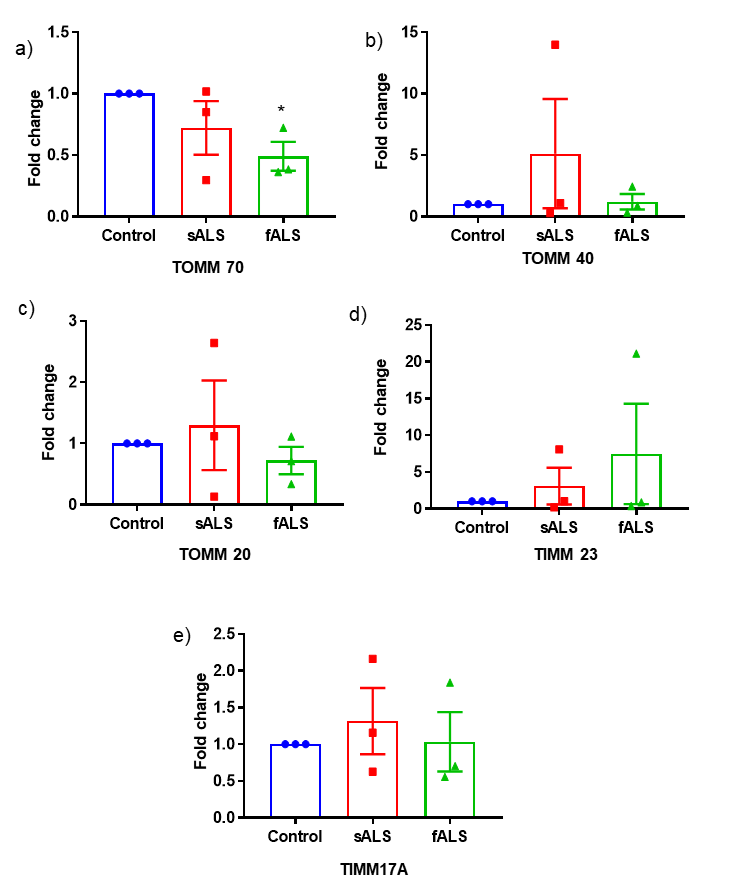


**Supplementary Figure 4:** Mitochondrial import complex transcription is unchanged in ALS iMNs. RNA was isolated from sALS, fALS, and control motor neuron samples and analyzed by qPCR with no changes identified except significantly decreased TOMM70 in fALS motor neurons (a-e). Gene expression was normalized to GAPDH and expressed relative to fold change values to control i-motor neurons. Data is shown n = 3. Each data point is the mean value for an individual line and the bar represents the mean of the three lines +/- SEM, *p<0.05 using Graphpad Prism version 7.03

**
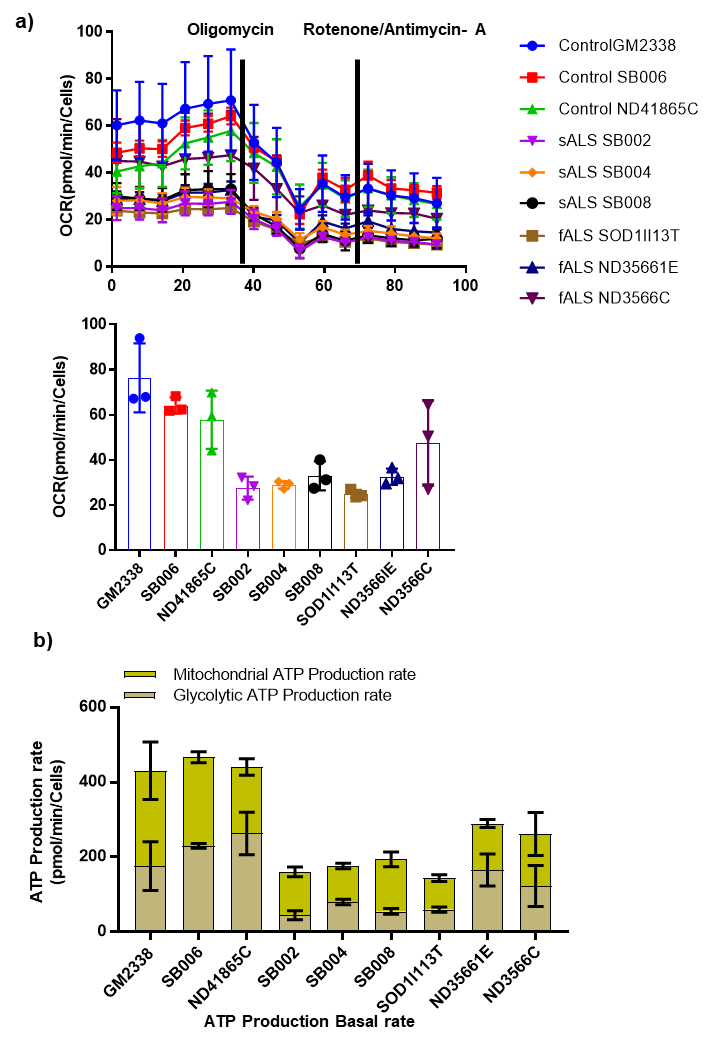
**

**Supplementary Figure 5:** Impaired energetics in ALS i-motor neurons for each unique patient-derived line: (a) Alterations in basal OCR and after treatment with oligomycin/rotenone in sALS & fALS i-motor neurons from each patient-derived line individually is demonstrated. Normalization for cell number was performed using nuclear stain. n=3, where each n is an independent differentiation into i-motor neurons per line, each of the 9 lines shown separately. b) Decreased ATP production (mitochondrial ATP and glycolytic ATP) in sALS & fALS motor neurons compared to control i-motor neurons after normalization using nuclear stain. Each data point is an independent differentiation of the line indicated, and the bar represents the mean of the three differentiations for each line +/- SEM.

**Table 1: Detailed information of materials used:**

| **REAGENT or RESOURCE** | | **SOURCE** | | | **IDENTIFIER** | | |
| --- | --- | --- | --- | --- | --- | --- | --- |
| **Antibodies** | | | | | | | |
| Rabbit polyclonal anti-VDAC | | Millipore-Sigma | | | Cat# AB10527 | | |
| Rabbit polyclonal anti-TIMM23 | | Abcam | | | Cat# ab116329 | | |
| Rabbit monoclonal anti-TIMM17A | | Abcam | | | Cat# ab192246 | | |
| Rabbit polyclonal anti-TOMM70A | | Abcam | | | Cat# ab83841 | | |
| Mouse monoclonal anti-TOMM40 | | Santa Cruz Biotechnology | | | Cat# D-2 SC365-467 | | |
| Rabbit monoclonal anti-TOMM20 | | Cell Signaling technology | | | Cat# 42406S | | |
| Total OXPHOS Rodent WB Antibody Cocktail (Mouse) | | Abcam | | | Cat# ab110413 | | |
| IRDye 800CW Goat anti-Rabbit | | Li-Cor | | | Cat# 926-32211 | | |
| IRDye 800CW Goat anti-Mouse | | Li-Cor | | | Cat# 926-32210 | | |
| IRDye 680LT Goat anti-Rabbit | | Li-Cor | | | Cat# 926-68021 | | |
| IRDye 680LT Goat anti-Mouse | | Li-Cor | | | Cat# 926-68020 | | |
| Stem Light Pluripotency kit | | Cell Signaling technology | | | Cat# 9656S | | |
| Goat polyclonal SOX1 | | R&D Systems | | | Cat# AF3369 | | |
| Mouse monoclonal PAX6 | | DSHB | | | RRID AB-528427 | | |
| Rabbit polyclonal anti-Nestin | | Millipore-Sigma | | | Cat# ABD69 | | |
| Mouse monoclonal anti-AFP (alpha 1 Fetoprotein) | | Abcam | | | Cat# ab3980 | | |
| Rabbit polyclonal brachyury | | Santa Cruz Biotechnology | | | Cat# sc-20109 | | |
| Mouse monoclonal HB9 | | DSHB | | | Cat# RRID AB-2145209 | | |
| Rabbit monoclonal β3-Tubulin | | Cell Signaling technology | | | Cat# 5568 | | |
| Rabbit polyclonal anti-Choline Acetyltransferase (ChAT) | | Millipore-Sigma | | | Cat# AB143 | | |
| Mouse monoclonal anti-Neurofilament H (SMI32) | | Biolegend/Covance | | | Cat# 801702/SMI32P | | |
| Mouse monoclonal anti-MAP2 | | Millipore-Sigma | | | Cat# MAB3418 | | |
| Mouse monoclonal anti-GFAP | | Millipore-Sigma | | | Cat# G3893 | | |
| Donkey anti-rabbit alexafluor secondary antibodies | | Invitrogen/Thermofisher scientific | | | Cat# A-212207,A-21206 | | |
| Donkey anti-mouse alexafluor secondary antibodies | | Invitrogen/Thermofisher scientific | | | Cat# A-21202, A-21203 | | |
| Alkaline Phosphatase Live Stain | | Invitrogen/Thermofisher scientific | | | Cat# A14353 | | |
| **Chemicals** | | | | | | | |
| DMEM | | GIBCO/Thermofisher scientific | | | Cat# 11965-092 | | |
| Knockout DMEM | | GIBCO/Thermofisher scientific | | | Cat# 10829018 | | |
| Knockout Serum replacement | | GIBCO/Thermofisher scientific | | | Cat# 10828010 | | |
| bFGF | | Peprotech | | | Cat# 100-18B | | |
| FBS | | GIBCO/Thermofisher scientific | | | Cat# 16000044 | | |
| Penicillin-Streptomycin | | GIBCO/Thermofisher scientific | | | Cat# 15140-122 | | |
| L-Glutamine | | GIBCO/Thermofisher scientific | | | Cat# 25030081 | | |
| Polybrene | | Millipore-Sigma | | | Cat# 107689 | | |
| mTeSR | | Stem cell Technologies | | | Cat# 85870 | | |
| Stemflex | | Stem cell Technologies | | | Cat# A33493-01 | | |
| ReLeSR | | Stem cell Technologies | | | Cat# 05872 | | |
| Stemdiff Neural Induction medium | | Stem cell Technologies | | | Cat# 05835 | | |
| AggreWell™ EB Formation Medium | | Stem cell Technologies | | | Cat# 05893 | | |
| Stemdiff Neural Progenitor medium | | Stem cell Technologies | | | Cat# 05833 | | |
| Accutase cell dissociation reagent | | Stem cell Technologies | | | Cat# 07920 | | |
| Gentle cell dissociation reagent | | Stem cell Technologies | | | Cat# 07174 | | |
| Neuraobasal medium | | Life Technologies | | | Cat# 21103049 | | |
| DMEM/F12 | | Life Technologies | | | Cat# 11330032 | | |
| B27 Supplement (w/o Vitamin A) | | Life Technologies | | | Cat# 12587010 | | |
| N2 Supplement | | Life Technologies | | | Cat# 17502048 | | |
| Glutamax | | Life Technologies | | | Cat# 35050-061 | | |
| 2-Mercaptoethanol | | Life Technologies | | | Cat# 21985023 | | |
| Matrigel | | Corning | | | Cat# 354277 | | |
| Laminin | | Stem cell Technologies | | | Cat# L2020 | | |
| Polyornithine | | Stem cell Technologies | | | Cat# P4957 | | |
| Ascorbic Acid | | Millipore-Sigma | | | Cat# A4544 | | |
| Retinoic Acid | | Millipore-Sigma | | | Cat#R2625 | | |
| LDN193189 | | Stemgent | | | Cat# 04-0074-02 | | |
| SB431542 | | Stem cell Technologies | | | Cat# 72232 | | |
| DAPT | | Stem cell Technologies | | | Cat# 72082 | | |
| CHIR99021 | | Tocris | | | Cat# 4423 | | |
| Y-27632 | | Tocris | | | Cat# 1254 | | |
| Smoothened Agonist (SAG 1.3) | | Stem cell Technologies | | | Cat# 73412 | | |
| BDNF | | Peprotech | | | Cat# 450-02 | | |
| GDNF | | Peprotech | | | Cat# 450-10 | | |
| CNTF | | Peprotech | | | Cat# 450-13 | | |
| Papain | | Worthington | | | Cat# LK003178 | | |
| DNase I | | Worthington | | | Cat# LK003172 | | |
| TMRM | | ThermoFisher | | | Cat#T668 | | |
| Mitosox | | ThermoFisher | | | Cat#M36008 | | |
| DCFDA | | Millipore-Sigma | | | Cat# D6883 | | |
| JC-1 Iodide | | Santacruz biotechnology | | | Cat#CAS47729-63-5 | | |
| Hoechst stain | | Millipore-Sigma | | | Cat#B2883 | | |
| TaqMan master mix | | ThermoFisher | | | Cat# 4304437 | | |
| PowerUP Sybr Green | | ThermoFisher | | | Cat# A25742 | | |
| Bolt™ 4-12% Bis-Tris Plus Gels | | ThermoFisher | | | Cat#NW04120BOX | | |
| RNeasy Kit | | Qiagen | | | Cat#74104 | | |
| High Capacity Reverse Transcription Kit | | ThermoFisher | | | Cat#4368814 | | |
| Mitochondrial isolation kit ,HUMAN | | Miltenyi Biotech | | | Cat#130-094-532 | | |
| XF Real-Time ATP Rate Assay Kit | | Agilent | | | Cat# No.: 103592–100 | | |
| **Oligonucleotide/Primers** | | | | | | | |
| GAPDH | Hs99999905_m1 | | | Invitrogen | | | |
| SOX2 | Hs00602736_s1 | | | Invitrogen | | | |
| NANOG | Hs02387400_g1 | | | Invitrogen | | | |
| POU5F1 | Hs03005111_g1 | | | Invitrogen | | | |
| NESTIN | FP; 5’- GTCTCAGGACAGTGCTGAGCCTTC-3’ | | | Sigma | | Custom preparation | |
|  | RP; 5’-TCCCCTGAGGACCAGGAGTCTC-3’ | | |  |  |  |  |
| SOX1 | FP; 5’-GAGATGATCAGCATGTACCTGCC-3’ | | | Sigma | | Custom preparation | |
|  | RP; 5’-GTAGTGCTGTGGCAGCGAGT-3’ | | |  |  |  |  |
| HB9 | FP; 5’-CCTAAGATGCCCGACTTCAA-3’ | | | Sigma | | Custom preparation | |
|  | RP; 5’-TTCTGTTTCTCCGCTTCCTG-3’ | | |  |  |  |  |
| CHAT | FP; 5’-ACTCCATTCCCACTGACTGTGC-3’ | | | Sigma | | Custom preparation | |
|  | RP; 5’-TCCAGGCATACAAGGCAGATG-3’ | | |  |  |  |  |
| PAX6 | FP; 5’-GCCCTCACAAACACCTACAG-3’ | | | Sigma | | Custom preparation | |
|  | RP; 5’-TCATAACTCCGCCCATTCAC-3’ | | |  |  |  |  |
| NEFM/SMI32 | FP; 5’- AGGACCTGCTCAATGTCAAG-3’ | | | Sigma | | Custom preparation | |
|  | RP; 5’- TGGACACAGAGGGAATTTTGG-3’ | | |  |  |  |  |
| TOMM70A | FP; 5’- ACTACGAGCTACCTTCTACCTG-3’ | | | Sigma | | Custom preparation | |
|  | RP; 5’- CATGCTGCCTCTTTTGATGAG-3’ | | |  |  |  |  |
| TOMM40 | FP; 5’- GCACTGTCATGTCTCTAGCTG-3’ | | | Sigma | | Custom preparation | |
|  | RP; 5’- CCTCAAACTCCACACCCAC-3’ | | |  |  |  |  |
| TOMM20 | FP; 5’- ACAGAAGCTTGCCAAGGAG-3’ | | | Sigma | | Custom preparation | |
|  | RP; 5’- CTACGCCCTTCTCATATTCACC-3’ | | |  |  |  |  |
| TIMM23 | FP; 5’- TCGGCTAGGATTGAAGGAAAC-3 | | | Sigma | | Custom preparation | |
|  | RP; 5’- GATGACACCAAATGCACTATAGAG-3’ | | |  |  |  |  |
| TIMM17A | FP; 5’- GGCATTGGAGTCAAGATGGAG-3’ | | | Sigma | | Custom preparation | |
|  | RP; 5’- CACTGGAGAATTGCGAAAACC-3’ | | |  |  |  |  |
| β-Actin | FP; 5’-ACCTTCTACAATGAGCTGCG-3’ | | | Sigma | | Custom preparation | |
|  | RP; 5’-CTGGATGGCTACGTACATGG-3’ | | |  |  |  |  |
| **Software and algorithms** | | | | | | | |
| ImageJ/Fiji | Girish and Vijayalakshmi, 2004; Schindelin et al., 2012 | | | Immunofluorescence | | | |
| Graphpad Prism 7.0 | Graphpad | | | Statistics | | | |
| Biorad CFX Manager | Biorad | | | qPCR | | | |
| Image Studio 2.1 | Licor Biosciences | | | Western Blotting | | | |
| Synergy H1 Plate reader | Biotek | | | ROS/MMP/LDH | | | |
| Personal Molecular Imager | Biorad | | | Import Assay | | | |
| **Cell lines** | | | | | | | |
| iPSC | Control/ALS | | Mutation | Sex | | | Karyotype |
| SB006 | Control | | N/A | Female | | | Normal |
| GM2338 | Control | | N/A | Male | | | Normal |
| ND41865C | Control | | N/A | Male | | | Normal |
| SB002 | sALS | | N/A | Male | | | Normal |
| SB004 | sALS | | N/A | Male | | | Normal |
| SB008 | sALS | | N/A | Female | | | Normal |
| ND35660C | fALS | | SOD1D90A | Female | | | Normal |
| ND3566IE | fALS | | SOD1I113T | Male | | | Normal |
| CS22iALS-SOD1I113Tn2 | fALS | | SOD1I113T | Male | | | Normal |

**Full gel image of mitochondrial protein Import images for Figure 3**

**
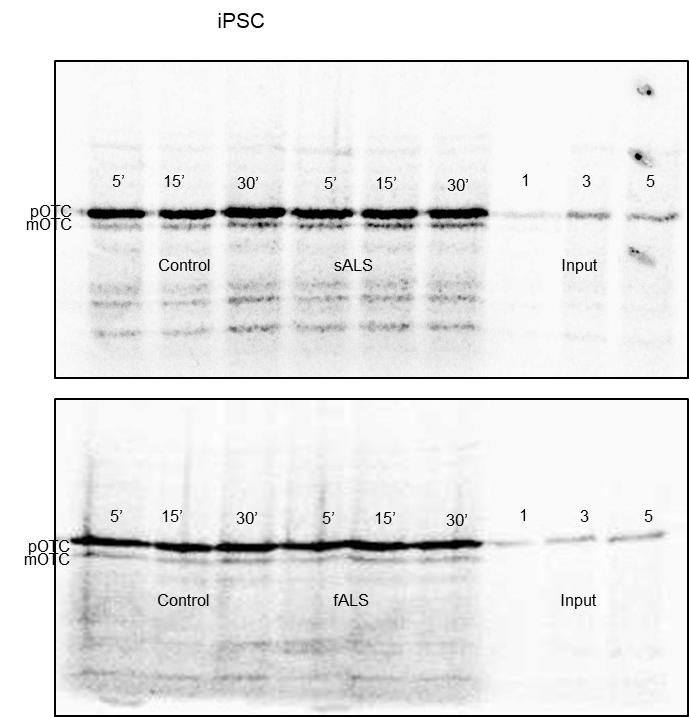
Figure 3a**

**
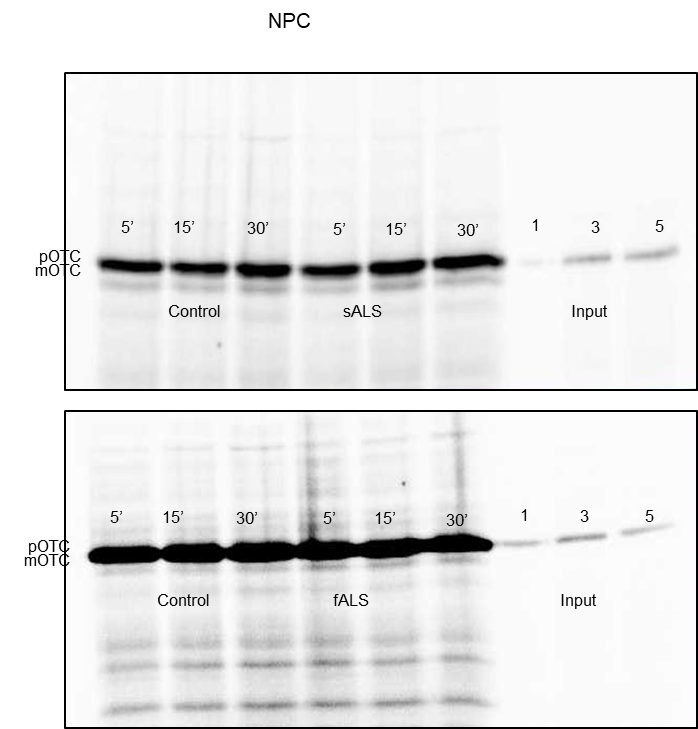
Figure 3b**

**
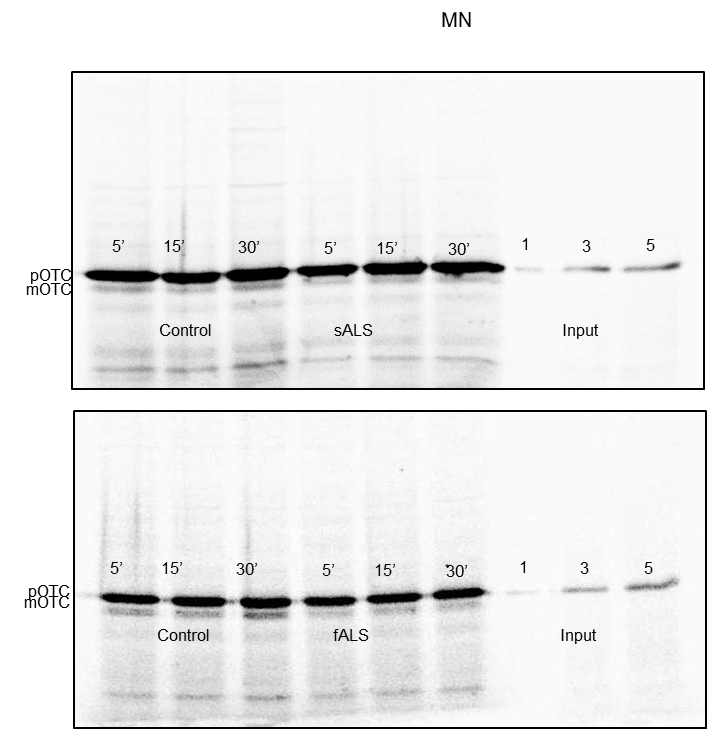
Figure 3c Full western blot images of mitochondrial import complex proteins for Figure 4a**

**
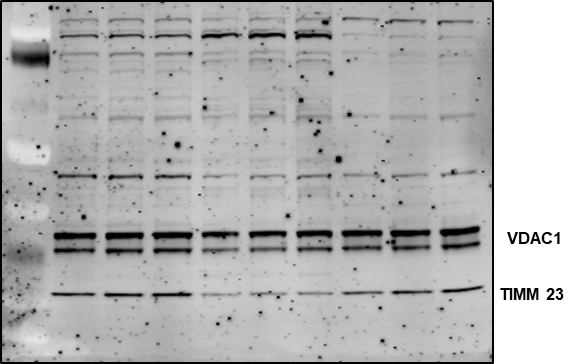

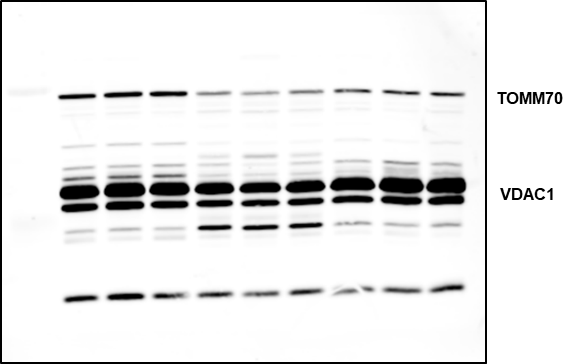

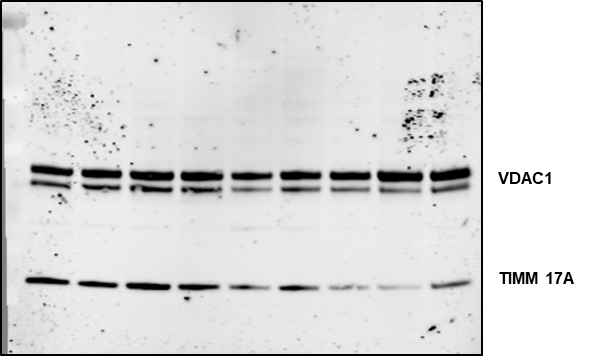
**

**
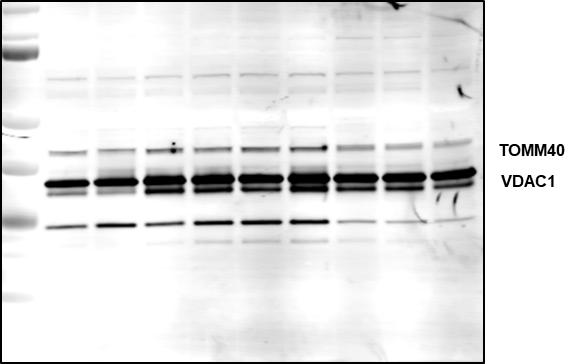
**

**
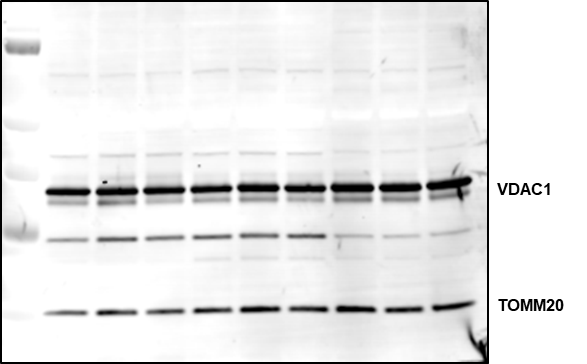
**

**Full western blot images of mitochondrial OXPHOS proteins (mitoprofiler) for Figure 5a**


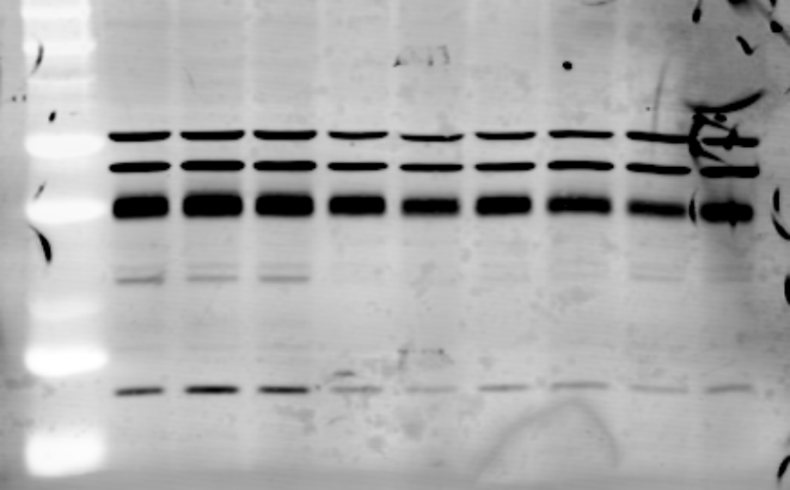

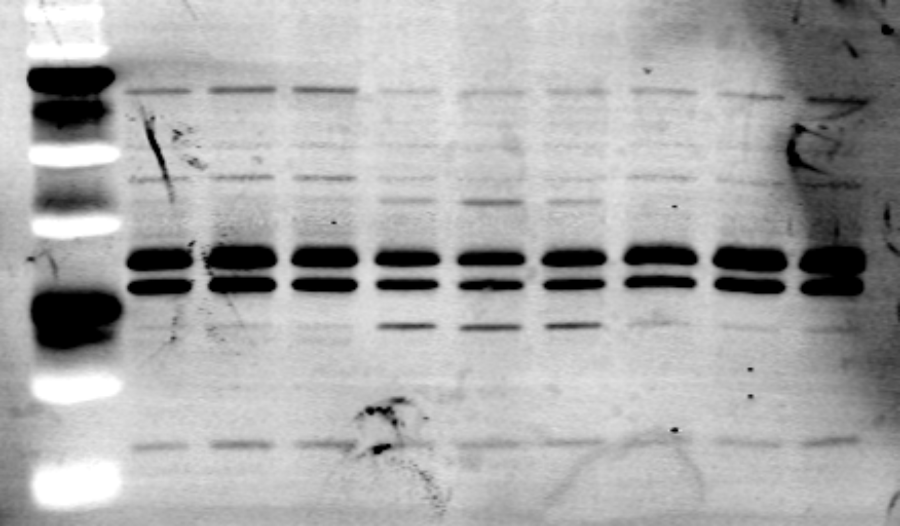


**Complex IV**

**Complex V**

**Complex III**

**Complex I**

**VDAC1**
